# Neuronal cell culture from transgenic zebrafish models of neurodegenerative disease

**DOI:** 10.1242/bio.036475

**Published:** 2018-09-06

**Authors:** Jamie R. Acosta, Maxinne Watchon, Kristy C. Yuan, Jennifer A. Fifita, Adam J. Svahn, Emily K. Don, Claire G. Winnick, Ian P. Blair, Garth A. Nicholson, Nicholas J. Cole, Claire Goldsbury, Angela S. Laird

**Affiliations:** 1 The Brain & Mind Centre, University of Sydney, Sydney, New South Wales 2050, Australia; 2 The Bosch Institute, University of Sydney, Sydney, New South Wales 2006, Australia; 3 Discipline of Anatomy and Histology, University of Sydney, Sydney, New South Wales 2006, Australia; 4 Sydney Medical School, University of Sydney, Sydney, New South Wales 2006, Australia; 5 Centre for Motor Neuron Disease Research, Department of Biomedical Sciences, Faculty of Medicine and Health Sciences, Macquarie University, Sydney, New South Wales 2109, Australia; 6 ANZAC Research Institute, Concord Repatriation Hospital, Sydney, New South Wales 2139, Australia

**Keywords:** Primary neuronal cell culture, Transgenic zebrafish, Amyotrophic lateral sclerosis (ALS), Spinocerebellar ataxia type-3, Fused in sarcoma (FUS), Ataxin-3 (ATXN3)

## Abstract

We describe a protocol for culturing neurons from transgenic zebrafish embryos to investigate the subcellular distribution and protein aggregation status of neurodegenerative disease-causing proteins. The utility of the protocol was demonstrated on cell cultures from zebrafish that transgenically express disease-causing variants of human fused in sarcoma (FUS) and ataxin-3 proteins, in order to study amyotrophic lateral sclerosis (ALS) and spinocerebellar ataxia type-3 (SCA3), respectively. A mixture of neuronal subtypes, including motor neurons, exhibited differentiation and neurite outgrowth in the cultures. As reported previously, mutant human FUS was found to be mislocalized from nuclei to the cytosol, mimicking the pathology seen in human ALS and the zebrafish FUS model. In contrast, neurons cultured from zebrafish expressing human ataxin-3 with disease-associated expanded polyQ repeats did not accumulate within nuclei in a manner often reported to occur in SCA3. Despite this, the subcellular localization of the human ataxin-3 protein seen in cell cultures was similar to that found in the SCA3 zebrafish themselves. The finding of similar protein localization and aggregation status in the neuronal cultures and corresponding transgenic zebrafish models confirms that this cell culture model is a useful tool for investigating the cell biology and proteinopathy signatures of mutant proteins for the study of neurodegenerative disease.

## Abstract (precis)

**Summary:** This article describes the optimization and validation of a protocol for culturing of neurons from transgenic zebrafish for the study of neurodegenerative diseases.

## INTRODUCTION

The zebrafish (*Danio rerio*) is increasingly used to successfully model neurodegenerative diseases (Babin et al., 2014; Bai and Burton, 2011; Bandmann and Burton, 2010; Bosco et al., 2010; Kabashi et al., 2010; Laird et al., 2016; Lemmens et al., 2007; McGown et al., 2013; Miller et al., 2005; Paquet et al., 2009; Ramesh et al., 2010) and holds promise for testing potential disease treatments (McGown et al., 2013; Schiffer et al., 2007). There are straightforward methods available for modulating gene expression in zebrafish (Don et al., 2017; Hruscha et al., 2013; Suster et al., 2009) and female zebrafish spawn large numbers of embryos making it possible to perform behavioral testing and drug study screens with relatively high throughput (Zon and Peterson, 2005). Many proteins associated with neurodegenerative disease in humans are homologous in zebrafish, highlighting potentially conserved molecular-cellular functions that can be readily investigated in the zebrafish model (Howe et al., 2013).

Zebrafish cells, including neural cells, can be cultured directly from developing embryos (Liu et al., 2010; Myhre and Pilgrim, 2010; Robles et al., 2011; Sakowski et al., 2012; Fassier et al., 2010; Ciarlo and Zon, 2016; Sassen et al., 2017). The potential of this method for investigating differentiated neurons has previously been achieved with later-stage embryos [>19 h post-fertilization (hpf)] (Sakowski et al., 2012). Here we focused on exploring the potential to study neurodegenerative diseases by applying and optimizing the technique using transgenic zebrafish expressing mutated forms of the proteins fused in sarcoma (FUS) and ataxin-3 to model amyotrophic lateral sclerosis (ALS) and spinocerebellar ataxia type-3 (SCA3), respectively. ALS is a fatal neurodegenerative disease that causes progressive paralysis due to loss of motor neurons within the brain and spinal cord. ALS can be caused by either non-inherited (sporadic) and/or inherited causes, with more than 25 different genes currently identified to be linked with the disease (Nguyen et al., 2018; Ling et al., 2013; Renton et al., 2014). One gene known to cause ALS is FUS (Vance et al., 2009). FUS is a ubiquitous, predominantly nuclear, multifunctional DNA- and RNA-binding protein (reviewed in Deng et al., 2014). More than 50 different FUS mutations have been discovered to cause familial ALS (fALS) (Vance et al., 2009; Deng et al., 2014; Kwiatkowski et al., 2009). SCA3 is a somewhat similar fatal neurodegenerative disease that results in gradual loss of control and coordination of muscles due to neuronal loss. The genetic cause of SCA3 is inheritance of an expanded CAG trinucleotide repeat region in the *ATXN3* gene (Costa Mdo and Paulson, 2012). Abnormal CAG nucleotide repeat expansions (>40 repeats) result in an ataxin-3 protein with a long polyglutamine (polyQ) repeat region that has multiple potential toxic effects (Costa Mdo and Paulson, 2012). We have recently reported that zebrafish expressing ataxin-3 with an expanded polyQ tract harbor disease hallmarks such as ataxin-3 positive cleavage fragments and impaired movement at 6 days post-fertilization (dpf) (Watchon et al., 2017).

Both ALS and SCA3 are characterized by the mislocalization, accumulation and aggregation of the respective mutated proteins in neurons, accompanied by neural cell dysfunction and death (Rub et al., 2013; Saberi et al., 2015). In this study, cell cultures derived from transgenic zebrafish larvae allowed investigation of the subcellular localization of mutated human FUS and ataxin-3 and the presence or absence of protein inclusions in different cell types, including differentiated neurons. We confirmed that the subcellular localization of the disease-causing proteins were the same in the cell cultures as in the living transgenic zebrafish for both models of neurodegenerative disease. These neuronal cell cultures, obtained from transgenic zebrafish lines of neurodegenerative diseases, have potential for use in drug screening assays for effectors of protein aggregation and mislocalization. In combination with zebrafish behavioral and physiological analysis, this is an additional tool to investigate the functional effects of cellular pathology in neurodegeneration.

## RESULTS

### Optimization of zebrafish neural cell cultures

**Fig. 1.**
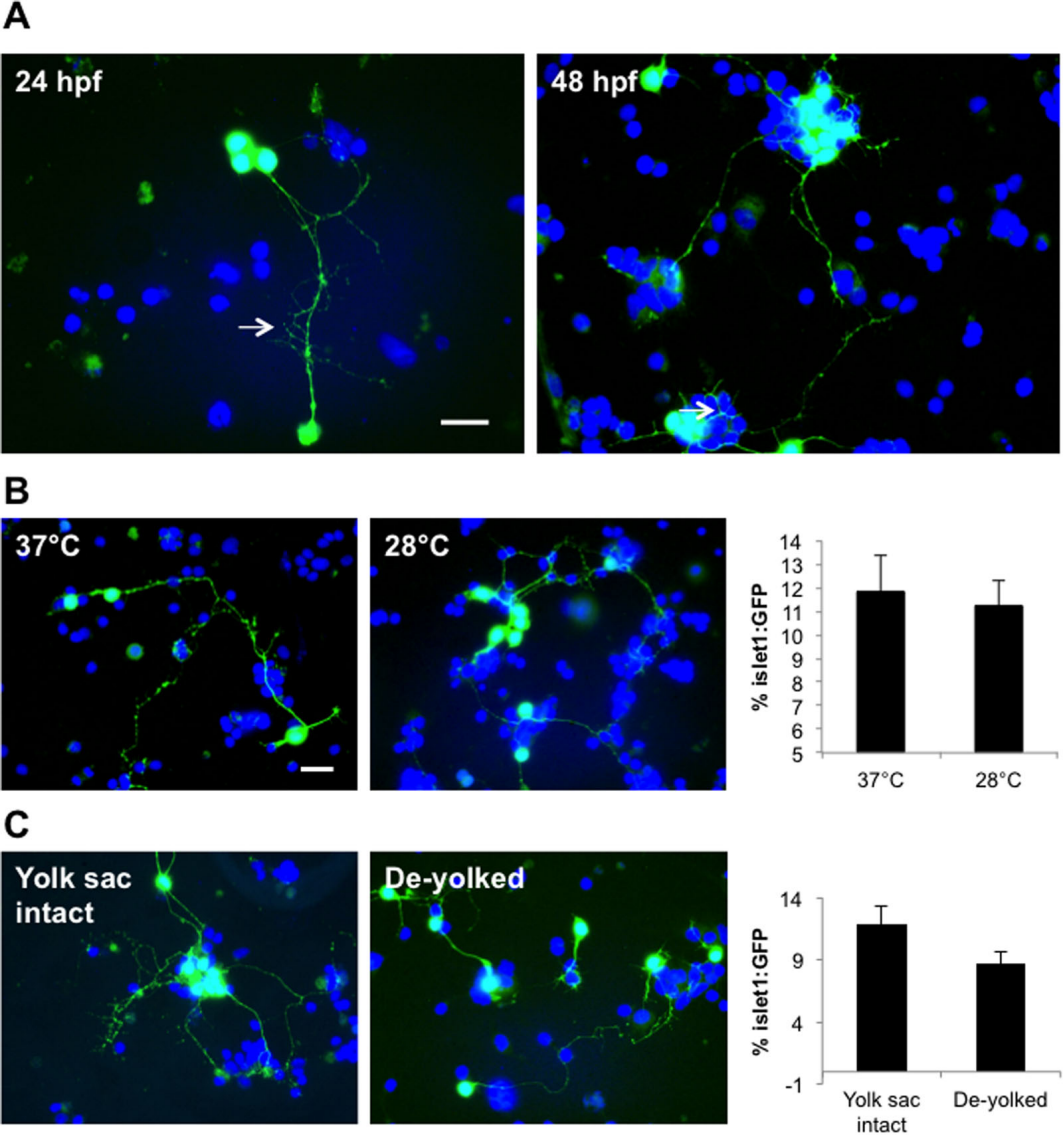
**Optimization of the zebrafish primary neural cell culture.** (A) Images of cell cultures derived from 24 hpf and 48 hpf-aged embryos. Motor neurons in both cultures exhibited outgrowth of long processes (arrows). (B) No difference in motor neuron survival rate was evident for cells incubated at 37°C or 28°C after 1 day (cultures from 24 hpf larvae). (C) Motor neurons in cultures derived from de-yolked embryos exhibited shorter neurites compared to those derived from whole embryos. Note however that by 2 div, almost 100% of cells from the de-yolked cultures were non-viable (not shown). Scale bar: 10 µm.

**Fig. 2.**
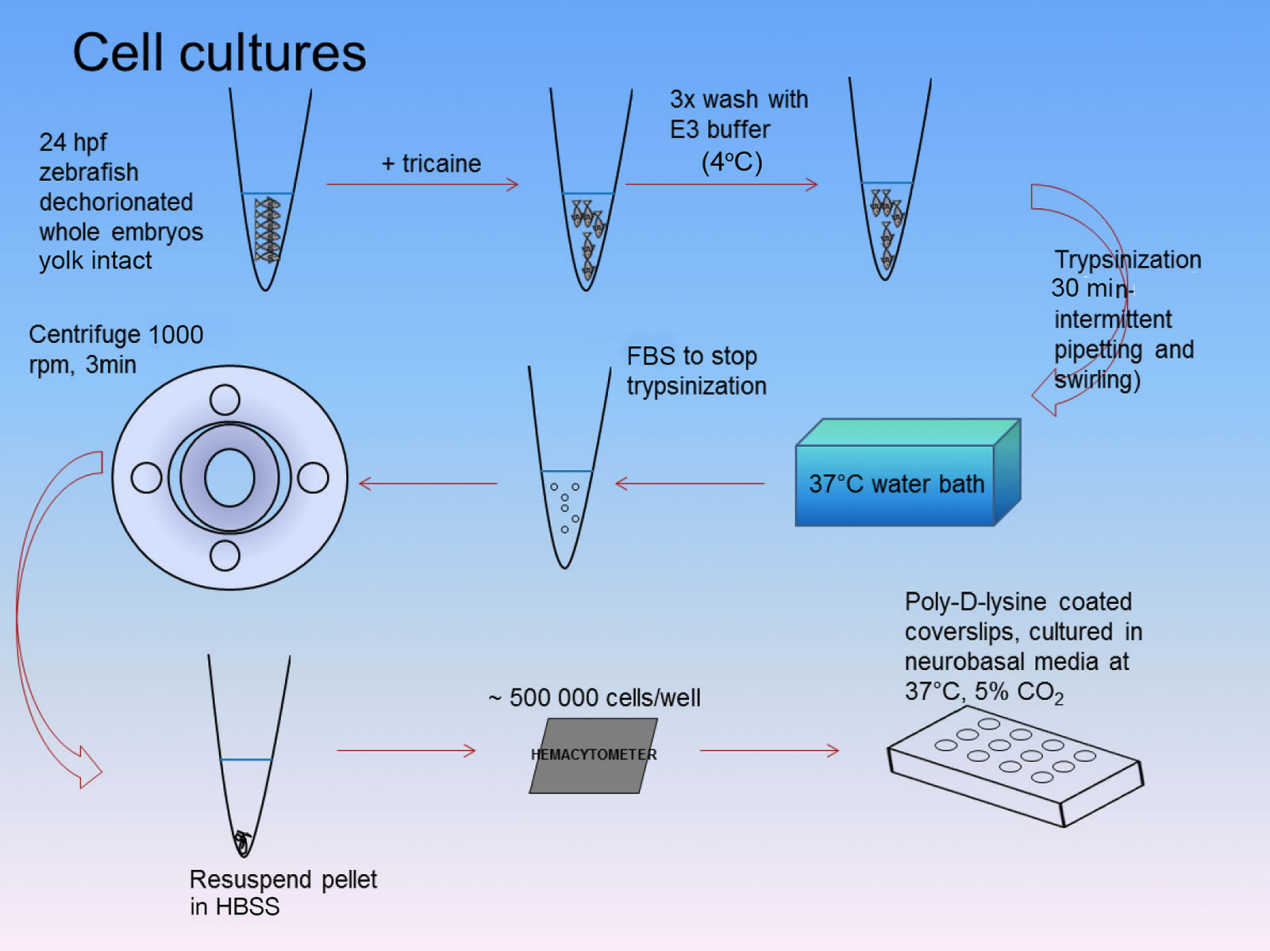
**Workflow for culturing zebrafish neurons.** Zebrafish embryos from 24 hpf or 48 hpf aged embryos were collected, dechorionated (with fine forceps) and placed into microtubes with E3 medium and 16 µM tricaine. Embryos were then washed three times with ice-cold E3 medium before being placed into 1× trypsin (in PBS) and pipetted intermittently for 30 min within a 37°C water bath. FBS was then added to stop dissociation and the tubes were then centrifuged for 3 min at 180 rcf (1000 rpm). The supernatant was removed and the cell pellet was resuspended in HBSS. Using a hemacytometer, approximately 500,000 cells were plated onto 12 mm coverslips pre-coated with poly-D-lysine and cultured in neurobasal media. Half this media was changed daily.

Cells harvested from transgenic zebrafish embryos expressing GFP in motor neurons under the *islet1* promoter (*islet1*:GFP) were used to generate primary zebrafish cell cultures and optimize the growth of neurons. From this, we determined the percentage of cells that expressed GFP to give us an indication of the degree of motor neuron survival. Embryos up to 48 hpf were cultured with ease, whilst embryos up to 96 hpf required longer incubations in trypsin to achieve cell dissociation, which was detrimental to subsequent cell survival. For this reason, we predominantly worked with cultures from 24 hpf zebrafish embryos. Cell cultures derived from both 24 and 48 hpf embryos maintained motor neuron integrity, with GFP positive neurons representing 10-12% of the total cells in culture and exhibiting rapid neurite outgrowth after 1 day *in vitro* (div; Fig. 1A). There was no difference in the percentage of GFP expressing cells or cellular morphologies when comparing cells grown at 28°C (controlled temperature in captivity) and 37°C (standard mammalian cell culturing temperature) (Fig. 1B), suggesting that both temperatures are suitable for culturing zebrafish motor neurons. In an attempt to improve the cell dissociation step we tested the effect of de-yolking the embryos by microsuction prior to culturing (Sakowski et al., 2012). We found that absence of the yolk gave rise to motor neurons with shorter neurites and widespread cell death after 2 div (although the percentage of motor neurons after 1 div remained unchanged relative to cultures from embryos with intact yolks) (Fig. 1C). In cultures derived from larvae with intact yolk sacs, motor neurons were viable for up to 1 week. A schematic representation of the optimized workflow required to derive these cultures is summarized in Fig. 2.

### Demonstration of mixed neural subtypes in culture

**Fig. 3.**
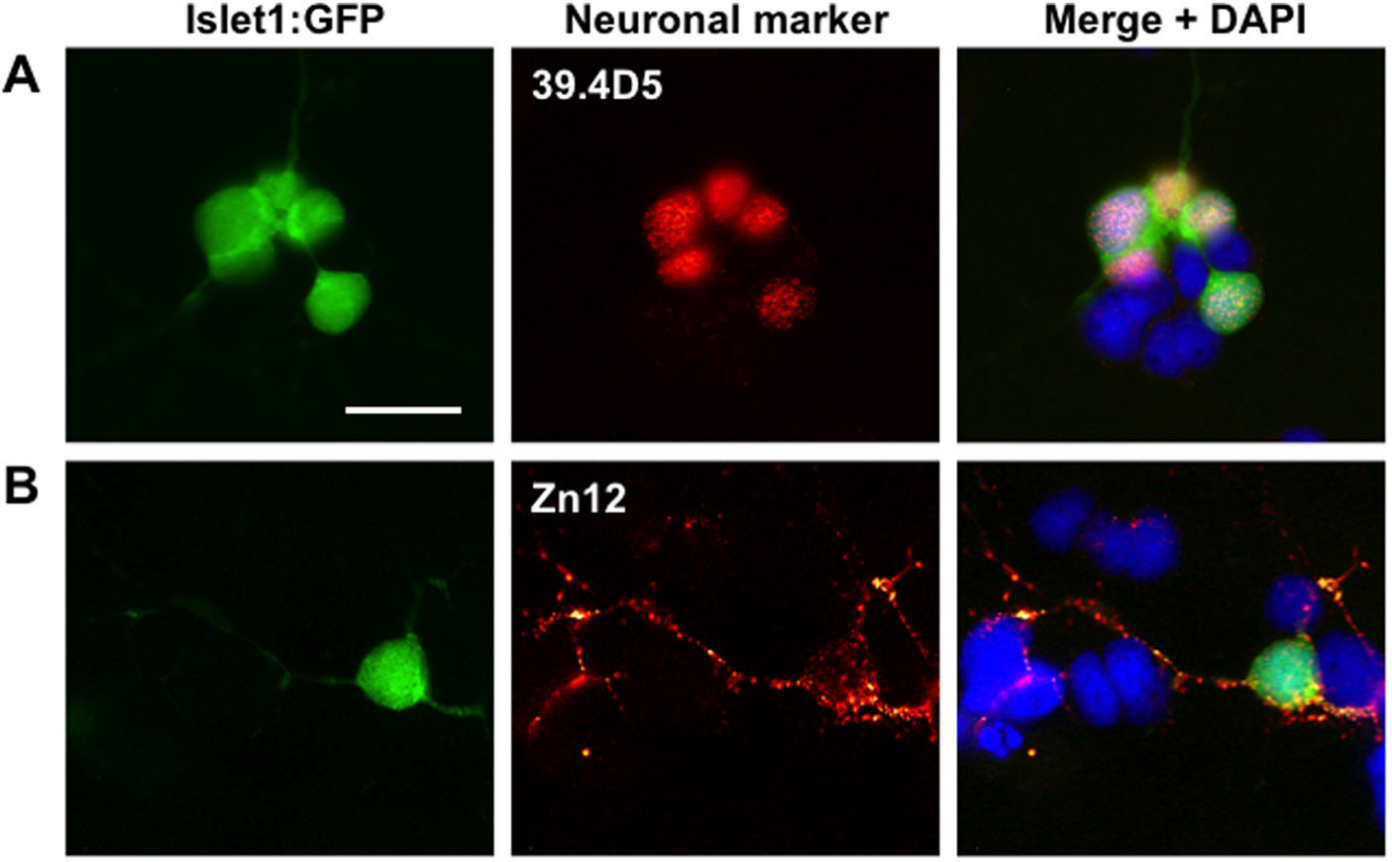
**Images of cultured 24 hpf Islet1:GFP zebrafish embryos stained with zebrafish-specific neuronal markers to confirm that the cell cultures contain various types of neurons.** (A) An Islet1:GFP motor neuron within the cultures is positively stained (red) for the neuronal marker 39.4D5 (islet1 and islet2 homeobox). (B) Another Islet1:GFP motor neuron, and nearby islet1:GFP negative cells, are stained positively (red) for the neuronal cell surface marker Zn12, indicating the inclusion of other types of neurons in addition to motor neurons. Scale bar: 10 µm.

As well as *islet1*:GFP-positive motor neurons, a variety of other neuronal subtypes were also present in the cultures, demonstrated by immunolabeling with neural cell antibodies obtained from the Developmental Studies Hybridoma Bank (University of Iowa). Anti-islet1/2 antibodies (39.4D5) labeled all GFP positive motor neurons as expected and additionally some GFP negative neurons presumably representing those expressing islet2 but not islet1 transcription factors (Fig. 3A). Some islet1 expressing cells and other cells were also labeled with by Zn12 antibodies against L2/HNK-1 carbohydrate epitope, a neural cell adhesion molecule expressed by a variety of different neural subtypes (Fig. 3B). In summary, a variety of motor neurons and other neural subtypes were evident in the mixed cultures.

### Culturing cells from transgenic zebrafish expressing pathogenic human motor neuron disease associated proteins

**Fig. 4.**
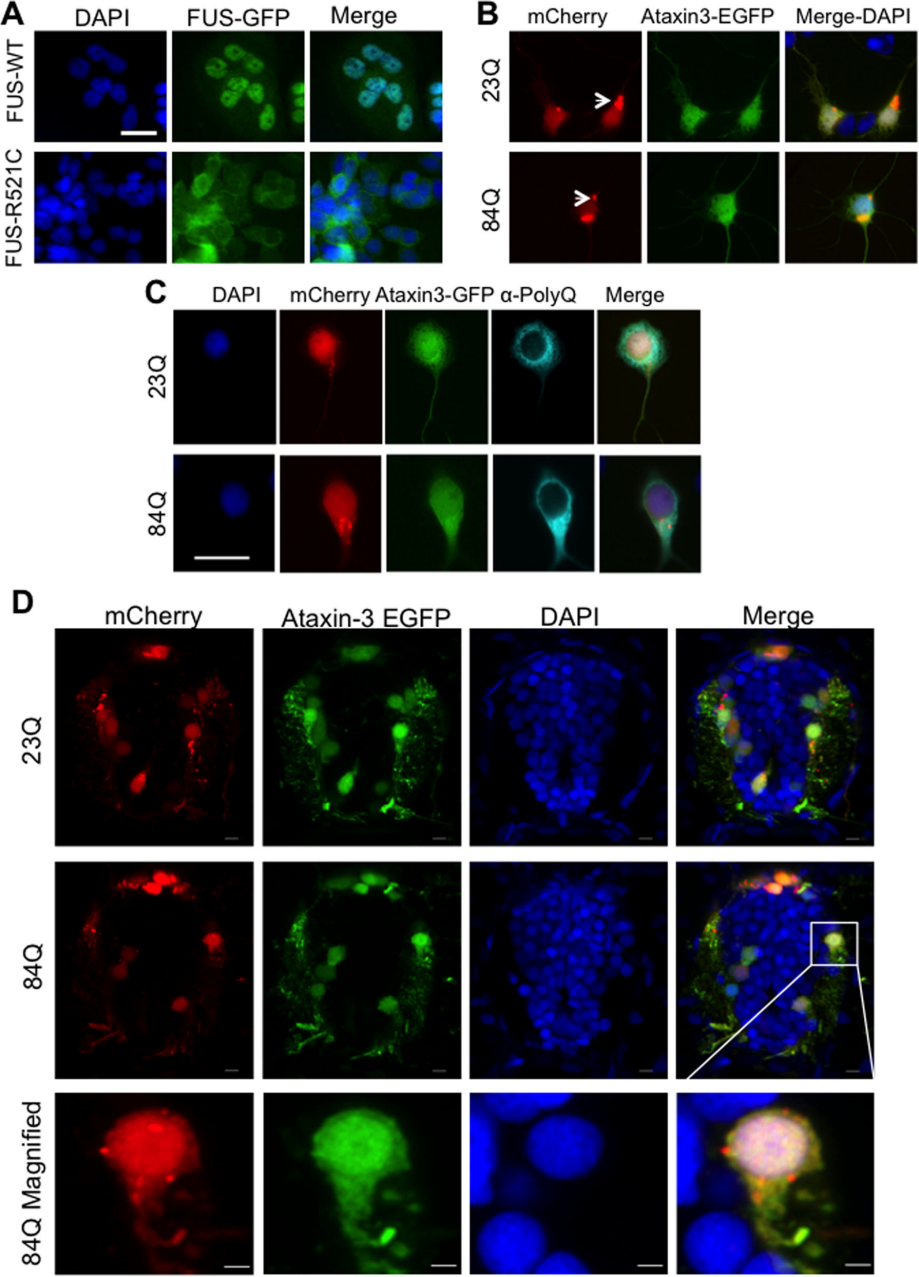
**Cultured cells derived from transgenic zebrafish larvae expressing neurodegenerative disease associated proteins FUS or ataxin-3.** (A) In cells cultured from mutant human FUS-GFP (FUS-R521C) zebrafish the FUS-GFP protein was mislocalized to the cytosol, whereas it remained predominantly nuclear in cells cultured from wild-type FUS-GFP zebrafish. (B) Cells cultured from double transgenic zebrafish expressing mCherry (red) and EGFP-ataxin-3-23Q/84Q (green) showed no obvious difference in fluorescent protein distribution in cells expressing non-pathogenic EGFP-ataxin-3-23Q and pathogenic EGFP-ataxin-3-84Q. Aggregates of mCherry-positive protein (arrows) were present in some neurons (Jakobs et al., 2000). (C) Immunolabeling cell cultures with anti-polyQ (pale blue) demonstrated cytosolic distribution of the ataxin-3 protein in cells expressing either EGFP-ataxin-3-23Q or pathogenic EGFP-ataxin-3-84Q. Scale bars: 10 µm. (D) Cross-sections of the spinal cord of 3 dpf transgenic SCA3 zebrafish revealed a similar expression pattern of EGFP-ataxin-3 and mCherry to that seen in the cell cultures. Scale bars: 5 µm.

We next cultured cells from transgenic zebrafish that expressed fluorescently tagged human neurodegenerative-disease related proteins (Fig. 4). In post-mortem tissue, cytosolic mislocalization and aggregation of FUS occurs in motor neurons (Kwiatkowski et al., 2009; Mackenzie et al., 2010). In our zebrafish cell cultures, fALS mutant human FUS (FUS-R521C) fused to GFP showed greater cytosolic distribution than wild-type human FUS (Fig. 4A). This is consistent with mislocalization seen in an FUS zebrafish model described previously (Acosta et al., 2014) and with other cell model studies (Dormann et al., 2010).

Cells were also cultured from a transgenic zebrafish model of SCA3 that express EGFP-fused to human ataxin-3 containing either a pathogenic (84Q) or non-pathogenic (23Q) polyglutamine tract. In the SCA3 zebrafish cell cultures, we did not detect any mislocalization or aggregation of EGFP-ataxin-3-84Q within the nucleus of neurons, a common phenotype reported in SCA3 patient brain and spinal cord samples (Rub et al., 2013). No major qualitative differences were present in the neurons from EGFP-ataxin-3-84Q zebrafish compared to non-pathogenic EGFP-ataxin-3-23Q zebrafish after 2 div (Fig. 4B). We did note some aggregation of the mCherry protein used as a neural cell reporter in our transgenic zebrafish, within cells cultured from both EGFP-ataxin-3-23Q and EGFP-ataxin-3-84Q zebrafish (Fig. 4B,C).

To confirm that the EGFP displayed in the cultured neural cells was indicative of the expression of EGFP-fused to human ataxin-3 we performed immunolabeling with a polyglutamine (polyQ) antibody (Fig. 4C). The polyQ staining pattern was found to be mostly cytoplasmic, and similar for the EGFP-ataxin-3-23Q and -84Q samples (Fig. 4C). This subcellular localization was consistent with that found in the live transgenic zebrafish expressing either EGFP-ataxin-3-23Q or -84Q at 3 dpf (Fig. 4D).

## DISCUSSION

Protocols exist for culturing cells from dissociated zebrafish embryos (Liu et al., 2010; Myhre and Pilgrim, 2010; Robles et al., 2011; Sakowski et al., 2012; Fassier et al., 2010; Ciarlo and Zon, 2016; Sassen et al., 2017; Andersen, 2001). A previous study demonstrated that differentiated zebrafish motor neurons can be cultured and maintained from embryos older than 19 hpf, up to 96 hpf, demonstrating the potential for this technique to be used to investigate the development and cell biology of motor neurons *in vitro* (Sakowski et al., 2012; Sassen et al., 2017). We adapted this method and used it to culture neurons from transgenic zebrafish models of neurodegenerative diseases. We show that zebrafish motor neurons grow neurites, differentiate and can be maintained in culture at either 28°C or 37°C. We found that de-yolking embryos prior to dissociation was detrimental to the survival of motor neurons in the cultures and led to stunted neurite outgrowth. This suggested the importance of endogenous factors and nutrients found in the yolk for growth and sustenance of differentiating neurons and other cell types (Carvalho and Heisenberg, 2010). Despite this, recent work in primary cell culture of zebrafish embryos has been successful in culturing a variety of neuronal cell types without the preservation of the yolk sac (Sassen et al., 2017). In previous work, selection of spinal neurons amongst a heterogeneous mixture of cells was achieved by performing larvae spinal cord dissections or fluorescent activated cell sorting (FACS) purification of neurons (Sakowski et al., 2012; Sassen et al., 2017). However, optimization of this particular protocol is required due to high mortality rates (Sassen et al., 2017). Enhanced motor neuron purity could enable investigation of cell-autonomous factors that have been shown to be important for the degenerative mechanisms of other motor neuron disease-related proteins such as superoxide dismutase-1 (SOD1) (Yamanaka et al., 2008). On the other hand, co-culturing motor neurons together with other cell types is biologically relevant and advantageous to cell survival due to available trophic factors in culture (Sassen et al., 2017). Similar to previous studies related to culturing zebrafish cells (Sakowski et al., 2012; Sassen et al., 2017), passaging of cells was not attempted.

Cultures from human mutant FUS transgenic zebrafish demonstrate that mutant, but not wild-type, human FUS is ubiquitously mislocalized in zebrafish cells, consistent with previous results in whole mount zebrafish larvae and cell cultures (Bosco et al., 2010; Acosta et al., 2014) and in mammalian cell lines (Bosco et al., 2010; Gal et al., 2011). This model offers another tool for gathering insight into mechanisms of FUS-linked disease. In contrast, we did not see any nuclear mislocalization or aggregation of expanded polyQ human ataxin-3 in the neurons cultured from our EGFP-ataxin-3 zebrafish larva (Watchon et al., 2017). Instead, the non-pathological EGFP-ataxin-3-23Q showed the same ataxin-3 distribution in zebrafish cells as the EGFP-ataxin-3-84Q protein. Ataxin-3 proteins with expanded polyQ stretches (>40Q repeats) are often reported to accumulate within the nucleus, often within inclusions, in SCA3 patient brain autopsy samples and in many animal models (Rub et al., 2013; Simoes et al., 2012). However, we did find aggregates of mCherry protein that did not localize with EGFP-ataxin-3. mCherry-positive protein aggregates have been reported previously, with red fluorescent proteins showing an increased propensity to self-aggregate when exposed to light (Jakobs et al., 2000). Nevertheless, these cell culture findings were consistent with what was seen in whole mount samples from the transgenic EGFP-ataxin-3 zebrafish larvae. This suggests that expanded polyQ ataxin-3, unlike human FUS, is not extensively mislocalized at this early age. Immunolabeling of transgenic zebrafish cells with ataxin-3 or polyQ specific antibodies confirmed that the exogenous protein and polyQ repeats maintained both nuclear and cytosolic distribution and did not accumulate within the nucleus. However, there was some nuclear GFP fluorescence not co-labeled with the polyglutamine antibody, suggesting partial degradation of the EGFP-ataxin-3 fusion protein, resulting in some GFP separated from the polyQ tract. These ataxin-3 expressing cell cultures provide an additional tool to complement the existing phenotypes already found in these SCA3 zebrafish (Watchon et al., 2017).

Overall, the protocol described here adds a new tool for investigating neurodegenerative diseases using zebrafish. We developed an easily adaptable method for the culturing of neurons from dissociated zebrafish embryos and further demonstrated ways of characterizing these cells *in vitro*. We provide examples of cultured cells that transgenically express proteins linked to the neurodegenerative diseases ALS and SCA3 and were able to demonstrate that the cultured cells maintained similar protein localization to the *in vivo* model from which they were generated. The zebrafish cell culture model offers another tool to gain insight into the molecular and cellular mechanisms underlying the diseases associated with these pathogenic proteins.

## MATERIALS AND METHODS

### Transgenic zebrafish

All experiments were carried out with approval from the University of Sydney Animal Ethics Committee (K00/3-2012/2/5709, K03/10-2010/3/5435 and K00/12-2010/3/5463) and Macquarie University (2016/04, 2015/034 and 2017/19). Transgenic zebrafish were bred on Tübingen/AB background and both male and female zebrafish were utilized. Transgenic zebrafish with GFP-expressing motor neurons driven by the *islet1* promoter Tg(*isl1*:GFP)rw0Tg are described in (Higashijima et al., 2000). Transgenic zebrafish Tg(*actb2*:Hsa.FUS-GFP)mq1Tg and Tg(*actb2*:Hsa.FUS_R521C-GFP)mq2Tg expressing human FUS conjugated to GFP driven by a β-actin promoter (called FUS-WT-GFP and FUS-R521C-GFP in the text) were generated as described previously (Acosta et al., 2014). Transgenic zebrafish Tg(*elavl3*:Gal4-VP16; mCherry); Tg(UAS:dsRed,EGFP-ATXN3_Q23) and Tg(*elavl3*:Gal4-VP16; mCherry); Tg(UAS:dsRed,EGFP-ATXN3_Q84) expressing EGFP tagged human ataxin-3 (containing either a normal polyQ motif [23Q] or a disease-linked expanded polyQ motif [84Q]) were generated using the UAS/Gal4 system as described by Watchon et al. (2017).

### Primary cell cultures

The cell culture protocol was based on a method developed by Sakowski et al. (2012) as well as other methodology used for the culturing of mammalian and avian neurons (Whiteman et al., 2011). Whole zebrafish embryos at 24 or 48 hpf were dechorionated manually with forceps, placed in microcentrifuge tubes and kept in ice-cold E3 medium with 16µM tricaine (anesthesia for zebrafish). These embryos were then washed multiple times with ice-cold sterile E3 medium and kept on ice for 20 min. For the experiment comparing cell cultures including or excluding the yolk sac, embryos were de-yolked by slow microsuction via syringe. Embryos were then dissociated in 1 x Trypsin diluted in PBS (Invitrogen) at 37°C within a water bath for 30 min with periodic gentle swirling and pipetting to aid dissociation. Trypsinization was arrested with DMEM supplemented with 10% FBS, L-alanyl-L-glutamine and antimycotic (Invitrogen). Cells were then pelleted by centrifugation at a relative centrifugal force of 180 (typically 1000 rpm) for 3 min. Coverslips were pre-coated with 0.1 mg/ml poly-D-lysine (Sigma-Aldrich) for 1 h and washed three times with PBS before plating. Cells were re-suspended in Hank's buffer salt solution (HBSS; Invitrogen) and plated at a density of 500,000 cells per 12 mm coverslip via a hemacytometer. Cells were placed in neuron-enriching Neurobasal™ media supplemented with 2% B27, L-alanyl-L-glutamine and antimycotic (Invitrogen). Multi-well plates were incubated at 37°C with 5% CO_2_ throughout the experiment (2 div) with half of the media replaced daily. For the experiments comparing the effect of temperature, plates containing cell cultures from transgenic zebrafish with the GFP-expressing motor neurons driven by the *islet1* promoter were incubated at either at 28°C or 37°C with 5% CO_2_ for 24 h. Cells were then fixed with 4% PFA in PBS and processed for immunofluorescent staining as described below.

### Immunofluorescence

Cells were fixed after 1 div for 15 min in 4% PFA in PBS pre-warmed to 37°C. Cells were then washed three times in PBS, permeabilized with 0.05% PBS/Triton-X-100 followed by another three PBS washes. Non-specific antibody binding was blocked by incubation by 5% goat serum (in PBS) prior to a 1 h incubation with one of the following primary antibodies: for islet 1 and islet 2 homeobox, 39.4D5 (1:50) and neuronal cell surface marker Zn12 (1:50), all obtained from the Developmental Studies Hybridoma Bank; for anti-polyglutamine (PolyQ) (Millipore, clone 5TF1-1C2|MAB1574). Coverslips were then washed in PBS and then incubated for 1 h with secondary antibodies Alexa Fluor 555 or 647 anti-mouse (1:200; Invitrogen, A32727 and A32728). Cells were counterstained with DAPI (1:1000; Sigma-Aldrich) and the coverslips were mounted in Prolong-Gold antifade reagent (Molecular Probes).

### Imaging and analysis

All cell cultures were imaged using a Zeiss Axio Observer inverted epifluorescence microscope equipped with a 40× Plan-Apochromat oil objective, xenon light source and Axiovision 4.8.2 acquisition software (Zeiss). Motor neuron density (per cent of islet1:GFP positive cells) from three independent experiments was calculated by the number of GFP-positive cells among the total number of DAPI-positive cells from ten images per coverslip taken from randomly selected regions (fragmented nuclei indicative of dead cells were not counted). Cross-section of the transgenic ataxin-3 zebrafish spinal cord at 3 dpf was imaged using the Leica TCS SP8 using a Leica HyD detector with a Leica 40× HC PL APO CS2 water immersion. DAPI was excited by a diode laser at 405 nm whilst the GFP and mCherry were excited by an OPSL laser at 488 nm and 552 nm respectively. Images were prepared with ImageJ 1.51w and adjusted by a mean filter with radius 1.

We compared the subcellular localization of the disease causing proteins FUS and ataxin-3 by comparing the localization of GFP expression with the DAPI stained nucleus.
